# Role of Podoplanin-Positive Cells in Cardiac Fibrosis and Angiogenesis After Ischemia

**DOI:** 10.3389/fphys.2021.667278

**Published:** 2021-04-12

**Authors:** Maria Cimini, Raj Kishore

**Affiliations:** Center for Translational Medicine, Lewis Katz School of Medicine, Temple University, Philadelphia, PA, United States

**Keywords:** podoplanin, fibrosis, pericyte, mesenchymal stem cells, telocyte

## Abstract

New insights into the cellular and extra-cellular composition of scar tissue after myocardial infarction (MI) have been identified. Recently, a heterogeneous podoplanin-expressing cell population has been associated with fibrogenic and inflammatory responses and lymphatic vessel growth during scar formation. Podoplanin is a mucin-like transmembrane glycoprotein that plays an important role in heart development, cell motility, tumorigenesis, and metastasis. In the adult mouse heart, podoplanin is expressed only by cardiac lymphatic endothelial cells; after MI, it is acquired with an unexpected heterogeneity by PDGFRα-, PDGFRβ-, and CD34-positive cells. Podoplanin may therefore represent a sign of activation of a cohort of progenitor cells during different phases of post-ischemic myocardial wound repair. Podoplanin binds to C-type lectin-like receptor 2 (CLEC-2) which is exclusively expressed by platelets and a variety of immune cells. CLEC-2 is upregulated in CD11b^high^ cells, including monocytes and macrophages, following inflammatory stimuli. We recently published that inhibition of the interaction between podoplanin-expressing cells and podoplanin-binding cells using podoplanin-neutralizing antibodies reduces but does not fully suppress inflammation post-MI while improving heart function and scar composition after ischemic injury. These data support an emerging and alternative mechanism of interactome in the heart that, when neutralized, leads to altered inflammatory response and preservation of cardiac function and structure. The overarching objective of this review is to assimilate and discuss the available evidence on the functional role of podoplanin-positive cells on cardiac fibrosis and remodeling. A detailed characterization of cell-to-cell interactions and paracrine signals between podoplanin-expressing cells and the other type of cells that compose the heart tissue is needed to open a new line of investigation extending beyond the known function of these cells. This review attempts to discuss the role and biology of podoplanin-positive cells in the context of cardiac injury, repair, and remodeling.

## Introduction

New discoveries during the last decades have challenged the existing scientific dogmas and provided new conceptual developments in a number of scientific topics. We experienced intense effort in studying organ regeneration with stem and progenitor cells: an area that has evolved with the advent of new investigative tools like single-cell sequencing and cellular lineage tracing in intact animals. In line with the growing knowledge on the post-MI cardiac responses, it is imperative to further detail the cellular composition and evolution of heart tissue after injury. In this regard, we (Cimini et al., 2017) characterized for the first time the presence of cells positive for a mucin-like transmembrane glycoprotein called podoplanin in the injured heart. These cells do not represent a new category of cells; on the contrary, podoplanin is co-expressed after injury by PDGFRα-, PDGFRβ-, and CD34-positive cells and continue to be expressed by lymphatic endothelial cells. The importance of *de novo* podoplanin expression comes from the fact that this glycoprotein is the only known ligand of C-type lectin-like receptor 2 (CLEC-2), highly expressed in platelets, activated monocytes, macrophages, and lymphocytes, and CLEC-2 signaling cascade contributes to the pro-inflammatory lineage of the immune cells (Table 1). Within the four different types of podoplanin co-expressing cells in injured heart described above, each specific group can be analyzed separately, although PDGFRα-, PDGFRβ-, CD34-positive cells and lymphatic endothelial cells have been collectively described to take part in regeneration, fibrosis, and inflammatory processes of the same pathologies. It is therefore meaningful to understand whether the already described activity of PDGFRα, PDGFRβ, and CD34 cells is similar when they express podoplanin, whether podoplanin indicates a different phenotype of these cells, or whether these cells acquire podoplanin in response to injury-induced inflammation. Thus, the co-expression of podoplanin may suggest the different roles these cells may play in homeostasis versus pathological conditions. In this review, we will provide a description of all the cell categories that express podoplanin, their role in tissue homeostasis, and the evolution of pathologies.

**TABLE 1:** Podoplanin-positive cells markers.

**Type of cell**	**Markers**								
	**LYVE-1**	**Prox-1**	**VEGFR3**	**PDGFRα**	**PDGFRβ**	**CD34**	**α-SMA**	**Vimentin**	**Podoplanin**
Podoplanin-positive cells	✓	✓	✓	✓	✓	✓			✓
Lymphatic endothelial cells	✓	✓	✓						✓
Pericytes				✓	✓	✓	✓	✓	✓
MSCs				✓	✓	✓		✓	✓
Mesenchymal stromal cells				✓	✓	✓		✓	✓
Telocytes				✓	✓	✓		✓	✓

*Podoplanin-positive cells positivity compared with lymphatic endothelial cells, pericytes, MSCs, mesenchymal stromal cells, and telocytes.*

## Podoplanin-Positive Cells in Disease and Homeostasis: Multiple Roles of One Glycoprotein

Podoplanin is a mucin-type, integral membrane glycoprotein also known as Aggrus, T1α, D2-40, gp36, and RANDAM-2. It is composed of 162 amino acid residues (43 kDa), the N-terminus is directed outside the cell, and the sequence is preserved within species (Ugorski et al., 2016). The intracellular domain is characterized by three basic amino acids responsible for binding to the ezrin-radixin-moesin complex (ERM), which is essential for cell migration. Within the extracellular domain, three adjacent tandem repeats of amino acid sequences directly bind the only known podoplanin receptor: C-type lectin-like receptor 2 (CLEC-2) (Nagae et al., 2014; Ugorski et al., 2016). CLEC-2 is highly expressed in platelets, dendritic cells, and activated monocytes and lymphocytes (Ugorski et al., 2016). Physiologically, podoplanin is expressed primarily on lymphatic endothelial cells, stromal cells of lymph nodes, type-I pneumocytes, and glomerular podocytes (Astarita et al., 2012, 2015). In addition, podoplanin expression was found in the epithelial lining of the coelomic wall of the pericardio-peritoneal canal, in the cell lining the pleural and pericardial cavity, and in the epicardium (Gittenberger-de Groot et al., 2007; Mahtab et al., 2008). Of late, podoplanin expression has been observed in a larger variety of cells but predominantly it determines the normal development of the lymphatic system, heart, and lung (Pan and Xia, 2015; Ugorski et al., 2016). During lymphangiogenesis, podoplanin-expressing cells from the cardinal vein bind CLEC-2 to platelets that aggregate to seal and separate the first lymphatic vessel from the cardinal vein (Pan and Xia, 2015). In heart development, podoplanin is fundamental for the epithelial-mesenchymal transition (EMT) of the pro-epicardial organ; it regulates the downregulation of E-cadherin, a process that allows epithelial cells to become mobile mesenchymal cells (Astarita et al., 2012). E-cadherin is downregulated by podoplanin in cancer cells and cancer-associated fibroblasts (CAFs), leading to invasive growth and metastasis (Mahtab et al., 2008; Ugorski et al., 2016).

### C-Type Lectin Receptors and Podoplanin Signaling

Podoplanin binds to the non-canonical side face of CLEC-2, a receptor that belongs to a large family of innate immunity receptors that share a structurally homologous carbohydrate recognition domain (Lepenies et al., 2013; Nagae et al., 2014; Suzuki-Inoue et al., 2017). Specifically, CLEC-2 belongs to Dectin-1 subfamily of C-type lectin receptors, and it has been characterized by an extracellular C-Type lectin-like domain and a single intracellular hemITAM motif that recruits spleen tyrosine kinase (Syk). CLEC-2 is highly expressed on platelets (Rayes et al., 2017), and its expression has been reported on CD11b^high^ (monocytes) and Gr-1^high^ myeloid cells (a lower level than platelets), dendritic cells, as well on a variety of leukocytes and neutrophils following inflammatory stimuli (Kerrigan et al., 2009; Chang et al., 2010; Lowe et al., 2015b; Suzuki-Inoue et al., 2017). Physiologically, the CLEC-2/podoplanin interaction is essential for the formation of the lymphatic system, the pro-epicardial organ, cerebrovascular patterning, and lymph node development and maintenance (Schacht et al., 2003; Hess et al., 2014; Suzuki-Inoue et al., 2017). CLEC-2 and podoplanin highly interact for the preservation of high endothelial venules in the lymph node, vascular integrity under inflammatory conditions, and the wound healing process (Suzuki-Inoue et al., 2017). Furthermore, the CLEC-2/podoplanin axis contribute to the generation of optimal adaptive immune responses (Astarita et al., 2012, 2015; Acton et al., 2014; Benezech et al., 2014; Suzuki-Inoue et al., 2017). In myeloid cells, Syk-dependent signaling activation through podoplanin can variably lead to the production of reactive oxygen species (ROS) and/or induction of innate immune genes, including pro-inflammatory cytokines due to the final activation of NFAT through Syk cascade (Mourao-Sa et al., 2011). In platelets, CLEC-2 induces tyrosine phosphorylation of the hemITAM motif and downstream signaling, leading to calcium mobilization and platelet aggregation (Rayes et al., 2017). Recently, the binding between podoplanin and CLEC-2 following activation of CLEC-2 positive cells has been described in many pathologies; mostly, the acquisition of podoplanin by mesenchymal cells and the interaction with immune cells have been highlighted. The unique and versatile binding modes between podoplanin and CLEC-2 open a new area of investigation in order to understand the consequences of this interaction especially in pathological and inflammatory conditions.

### Podoplanin in Tumor Biology

Podoplanin represents a marker of the major solid tumors, unfortunately with adverse prognosis and CAFs; it is also the master regulator of the cancer invasiveness due to the EMT-mediated cell migration and invasion. Podoplanin binding to ERM in cancer cells lead to RhoA-associated kinase-dependent ERM phosphorylation with a consequent mobilization of both cancer cells and CAFs (Fernandez-Munoz et al., 2011). On the other hand, podoplanin mediates the remodeling of the actin cytoskeleton in the absence of EMT by filopodia formation or invadopodia stability via the downregulation of the activities of small Rho family GTPases (Martin-Villar et al., 2015) or binding CLEC-2 on platelets and skipping the immune checkpoints. Podoplanin facilitates thrombus formation; in fact, tumor cells induce platelet aggregation, which protects cancer cells from sheer stress and host immunological defense. This phenomenon results in increased tumor growth and enhanced metastatic potential of the tumors (Pula et al., 2013). Based on the importance and relevance of podoplanin in tumor biology, antibody-based immunotherapies, and antagonist that suppress podoplanin/CLEC-2 binding and following platelet aggregation and cancer metastasis have been developed (Kaneko et al., 2006, 2012; Kato et al., 2006; Ogasawara et al., 2008; Nakazawa et al., 2011; Fujita and Takagi, 2012; Takagi et al., 2013, 2014; Kato and Kaneko, 2014; Miyata et al., 2014; Chang Y. W. et al., 2015; Rayes et al., 2017; Krishnan et al., 2018). The efficacy of antibody-based immunotherapy is due to the activation of apoptosis, antibody-dependent and complement-dependent cellular cytotoxicity, or simply neutralizing the binding between ligand and receptor or a protein with a complement motif (Macor et al., 2015). Besides cell-to-cell or cell-to-ERM interaction, cancer cells and podoplanin-positive CAFs release extra-cellular vesicles and exosomes that contain podoplanin mRNA and protein (Carrasco-Ramirez et al., 2016). Exosomes containing podoplanin promoted lymphatic vessel formation, EMT, upregulation of oncogenic protein, and diminished expression of tumor suppressors (Carrasco-Ramirez et al., 2016).

### Podoplanin as a Key Facilitator of Stromal and Immune Cell Interaction

The podoplanin/CLEC-2 axis takes place not only *in utero* and cancer biology (Suzuki-Inoue et al., 2017); but they interact with each other under several pathological conditions since CLEC-2 expression has been reported on circulating CD11b positive cells, dendritic cells, and a variety of leukocytes and neutrophils in basal conditions and following inflammatory stimuli (Mourao-Sa et al., 2011; Lepenies et al., 2013; Yan et al., 2013; Lowe et al., 2015b). On the other hand, interstitial stromal cells acquire podoplanin after organ injury (Acton et al., 2012; Ugorski et al., 2016). Specifically, mesenchymal stromal cells upregulate podoplanin at sites of infection and chronic inflammation; functionally podoplanin enables the interaction with platelets, aggregation, and formation of microthrombi alongside the mesenchymal stromal cell migration capacity (Ward et al., 2019). It is known that mesenchymal stromal cells and interstitial stromal cells acquire podoplanin under interferon-γ, transforming growth factor-β and tumor necrosis factor-α stimuli, but the full mechanism behind the expression of this glycoprotein is still unknown (Kunita et al., 2018). The expression of podoplanin can be considered as involvement of mesenchymal cells in the inflammatory reaction since the receptor, CLEC-2, is highly expressed on activated immune cells.

### Podoplanin in the Injured Heart

In the heart, the expression of podoplanin by interstitial cells was described for the first time by Cimini et al. (2017). They reported that podoplanin is expressed by a heterogeneous population of lymphangiogenic, fibrogenic, and mesenchymal progenitor cells (Cimini et al., 2017). In the adult heart, podoplanin-positive cells are rare, constituting less than 5% of the myocardial small cell population (Pinto et al., 2016). In fact, in homeostatic conditions, podoplanin is expressed only by cardiac lymphatic endothelial cells (Cimini et al., 2017). Cimini et al. (2017) analyzed the spatial and temporal distribution of the cells that acquire this glycoprotein after ischemic injury, and although podoplanin is a common lymphoendothelial marker, it is expressed with an unexpected heterogeneity and the appearance of podoplanin-positive cells increases over time from the acute (2 days) to the chronic phase of the myocardial infarction (MI; 2 weeks and 1 month). The interstitial podoplanin-positive cells did not express LYVE-1, a specific lymphatic endothelial marker, Prox-1, a major transcription factor of the lymphatic endothelial fate, and VEGFR-3 unless organized in cardiac lymphatic vessels (Brakenhielm and Alitalo, 2019). This suggests that a large portion of podoplanin-positive cells do not possess a differentiated lymphatic endothelial phenotype. Additionally, podoplanin-positive cells do not express markers of mature endothelial cells like CD31 and VEGFR2 (Loukas et al., 2011). The immunohistochemistry and the flow cytometry analysis of the infarcted hearts at different time points after MI showed that the podoplanin-positive cells were distinctly PDGFRα positive. The co-localization of PDGFRβ and CD34 with podoplanin was infrequent early after MI and strongly elevated at later stages of infarct healing in the mature scar. Since PDGFRα and CD34 are associated with the properties of immature mesenchymal cells and PDGFRβ is a marker of pericytes the concordance of co-staining with podoplanin suggested that podoplanin-expressing cells contain also a population with progenitor capabilities. Podoplanin-positive cells are positive only for CD34 but negative for CD45, which exclude the hematopoietic origin. Of note, although PDGFRα and PDGFRβ are also associated with fibrogenic behavior, podoplanin-positive cells do not express vimentin and α-Smooth muscle actin at any time point, suggesting that podoplanin-positive cells do not generate fully differentiated fibroblasts (Cimini et al., 2017). Therefore, podoplanin may represent a sign of activation of a cohort of cells during different phases of postischemic myocardial wound repair and it can be involved in mechanisms of inflammation and scar formation after MI. Cimini et al. (2019) investigated a neutralizing antibody treatment approach in a mouse MI model to inhibit cell-to-cell interaction of podoplanin-positive cells to inflammatory cells in the modulation of post-MI inflammation since an exacerbated and prolonged inflammatory response is the leading cause of adverse remodeling after myocardial injury (Prabhu and Frangogiannis, 2016; Frangogiannis, 2017) and reported improved cardiac function after MI with this approach. Targeted anti-inflammatory approaches were widely studied to reduce inflammation and improve cardiac repair (Frangogiannis, 2012, 2014); although, patients treated with highly selective strategies did not show a positive outcome after therapy (Saxena et al., 2016). The translational failure may be the result of exclusive inhibition of the recruitment of pro-inflammatory monocytes and decreased cytokine expression in the ischemic hearts (Saxena et al., 2016). The complete suppression of the inflammatory pathways interferes with the migration and activation of reparative and regenerative cells important for positive tissue remodeling (Saxena et al., 2016). Cimini et al. (2019) demonstrated that inhibition of the interaction between podoplanin-expressing cells and podoplanin-binding cells reduce but does not fully suppress the inflammation post MI and at the same time enhance an endogenous myocardial regeneration process after ischemic injury (Cimini et al., 2019). The histological data, vis-a-vis the functional one, demonstrated that the neutralizing activity of podoplanin leads to the healthier tissue geometry organization in the treated animals compared to the scar formation in the control animals; enhanced cardiac performance, regeneration and angiogenesis post MI (Cimini et al., 2019). Modulating the interaction between podoplanin-positive cells and immune cells post MI positively affect the immune cells recruitment (Tugal et al., 2013; Frangogiannis, 2015; Roszer, 2015; Sica et al., 2015; Sager et al., 2017) and they did not observe any differences in podoplanin expression in acute and chronic phases after MI between the treated and untreated groups; the treatment with the neutralizing antibody does not affect the cell migration or the cell composition in the scar, thus, podoplanin-expressing cells resident in the heart displayed podoplanin under inflammatory condition and the neutralizing antibody could only interfere in the interaction of podoplanin with podoplanin-binding cells (Cimini et al., 2019).

### Podoplanin in Vascular Pathophysiology

Similar observations were reported by other groups studying the biology of inflammation and possible selective targets in several pathologies. Specifically, in vascular biology, CLEC-2 is known to maintain the physiological state of blood vasculature under inflammatory conditions; mice with a deficiency in CLEC-2 as well as inhibition of podoplanin are protected against deep vein thrombosis with reduced platelet accumulation at the inferior vena cava (IVC) wall (Payne et al., 2017). Podoplanin was found in the IVC wall and was localized in the vicinity of the abluminal side of the endothelium in an animal model of deep vein thrombosis (Payne et al., 2017) or in aspirated coronary thrombi (lytic and organized) from a patient with ST- elevation myocardial infarction (Rakocevic et al., 2016). The level of podoplanin in the IVC increased after 48 h of stenosis to a substantially higher extent in mice with a thrombus versus those without a thrombus. Treatment of animals with an anti-podoplanin neutralizing antibody resulted in the development of smaller thrombi; thus, Payne et al. (2017) proposed a novel mechanism of deep vein thrombosis where CLEC-2 and the upregulation of podoplanin trigger the thrombus formation. Platelets form stable aggregates on mouse podoplanin at arterial shear through the CLEC-2 pathway, and podoplanin thus supports platelet capture and activation at arteriolar rates of shear (Lombard et al., 2018). Consistent with the expression of podoplanin in thrombi, this glycoprotein is highly relevant in the calcification of aortic valves (Napankangas et al., 2019) and atherosclerotic lesions (Hatakeyama et al., 2012). Podoplanin is critical in the early stages of osteoblast-to-osteocyte transition (Ikpegbu et al., 2018) and, as already mentioned is acquired by mesenchymal stromal cells (Kunita et al., 2018). During osteogenesis fibroblast growth factor-2 promotes osteocyte differentiation and podoplanin expression (Ikpegbu et al., 2018). The calcification of aortic valves origin from mesenchymal cells which have differentiated toward an osteoblastic phenotype; immunohistochemical analysis of human calcified valves showed podoplanin positivity in lymphatic vessels, osteoblast, osteocyte, chondrocytes, macrophages, and spindle cells with a myofibroblastic phenotype (Napankangas et al., 2019). As well as for the calcification of aortic valves, podoplanin expression contributes to the thrombotic property of atherosclerotic lesions and might be a novel target for an anti-thrombus drug since it is highly co-localized with smooth muscle cells and macrophages in plaque with necrotic core compared with the early lesions composed by smooth muscle cells and small number of monocytes (Hatakeyama et al., 2012).

### Podoplanin in Neuropathology

Podoplanin is also investigated in neurobiology. It is expressed on the developing neural tube and neuro epithelium and guides the maturation and integrity of developing vasculature in the brain (Lowe et al., 2015a). In the adult brain it is highly expressed in a subset of glial fibrillary acidic protein-positive astrocytes adjacent to gliomas (Kolar et al., 2015); is co-expressed with nestin, a marker of neural progenitor cells; and has been suggested to be a marker for reactive astrocytes (Kolar et al., 2015). In lipopolysaccharide-induced neuroinflammation, podoplanin is expressed in neurons but not in astrocytes with a concomitant upregulation of active caspase 3, cyclin D1, and CDK4, which decreased *in vivo* and *in vitro* after knocking down podoplanin by siRNA (Song et al., 2014). As is the case with MI, in a mouse model of ischemic stroke with a middle cerebral artery occlusion, the expression of CLEC-2 and podoplanin increased after ischemia/reperfusion injury, with a peak at 24 h post-injury (Meng et al., 2020). Podoplanin and CLEC-2 colocalized mainly in the ischemia/reperfusion cortex and were expressed in neurons and microglia. Anti-podoplanin antibody pretreatment drastically reduced the cerebral infarct and attenuated the neurological deficits during the acute stage of recovery; moreover, a significant decrease of IL-18 and IL-1β was observed in mice pretreated with podoplanin neutralizing antibody (Meng et al., 2020). With this study, Meng et al. demonstrated that, like in the heart, the podoplanin/CLEC-2 axis plays an important role during inflammatory reactions (Cimini et al., 2019; Meng et al., 2020).

### Podoplanin and Autoimmune Diseases

Podoplanin is also well studied in the biology of autoimmune diseases, specifically psoriasis and rheumatoid arthritis (Noack et al., 2016a,b). During psoriasis mainly T helper lymphocytes infiltrate the inflammatory site and interact with mesenchymal cells and fibroblast, enhancing the production of IL-8, IL-6, and IL-1β; but, within all the activated peripheral blood mononuclear cells, monocytes contribute to higher IL-17 secretion and podoplanin expressing mesenchymal cells, largely contributes to this massive secretion (Noack et al., 2016b). Using an anti podoplanin antibody, the interaction with activated monocytes and mesenchymal cells inhibited IL-17 secretion by 60% (Noack et al., 2016b). A similar mechanism has been investigated within synoviocytes and activated peripheral blood mononuclear cells during rheumatoid arthritis (Noack et al., 2016a). Co-culture of peripheral blood mononuclear cells and synoviocytes highly increased the production of IL-6, IL-1β, and IL-17. Using podoplanin-neutralizing antibody during the co-culture reduced IL-17 secretion by 60%, inhibiting the binding between synoviocytes podoplanin positive and CLEC-2 positive activated monocytes (Noack et al., 2016a). These results lead to consider podoplanin as a potential target for chronic autoimmune diseases.

### Podoplanin in Organ Injury

Recently, podoplanin has been investigated also in pancreatic injuries; a different type of insult from inflammation or cancer may change the equilibrium in the tissue. In fact, podoplanin and Prox-1 expression is highly increased in pancreatic islets after a hypercaloric diet (Taran et al., 2019). In a mouse model of 6 weeks of hypercaloric diet-induced hypertrophy of pancreatic islets with a focal expression of podoplanin and Prox-1, at 9 weeks on a hypercaloric diet, strong peri-insular inflammation was found around the hypertrophic islets, highly expressing podoplanin, suggesting that podoplanin may be involved in the early steps of pancreatic islet changes (Taran et al., 2019).

### Podoplanin and New Insight Into Therapeutic Strategies

Taking together, neutralizing antibodies, antagonists, synthetic compounds, and CAR-T cells can inhibit podoplanin/CLEC-2 binding; and in all the pathologies where podoplanin is overexpressed any of these treatments may regulate the podoplanin function and improve the prognosis. Therefore, the contribution of podoplanin-positive cells in different pathologies must be fully investigated to generate information that goes beyond the cell-to-cell interaction and may involve also paracrine signals since the majority of podoplanin-positive cells have been described to release extracellular vesicles and exosomes (Krishnan et al., 2018).

## Lymphatic Endothelial Cells and Podoplanin

Based on the evidence from several research areas, podoplanin is acquired, specifically during inflammation, by a variety of cell types that do not express this glycoprotein at the baseline level, enhancing the inflammatory reaction mostly binding monocytes and platelets, and probably through paracrine factors. Which type of cells are resident podoplanin-positive cells in the heart? Looking overall at the markers of podoplanin-positive cells, few categories of well-known cells can be investigated. During physiological conditions podoplanin is expressed in the heart only by lymphatic endothelial cells (Pinto et al., 2016; Cimini et al., 2017); the cardiac lymphatic vasculature has been extensively investigated and studied (Brakenhielm and Alitalo, 2019) since an increase in lymphatics accompanied major cardiac pathological remodeling, such as acute and chronic ischemia, progressive atherosclerosis and hypertrophy, and myocarditis (Kholova et al., 2011). The lymphatic vasculature accompanies the blood vasculature, and it is essential for the maintenance of tissue fluid homeostasis and immune cell trafficking, specifically during and after a major injury (Brakenhielm and Alitalo, 2019). For this reason, many pharmacological treatments led to improved lymphangiogenesis (Yoon et al., 2003; Henri et al., 2016; Vieira et al., 2018) and thus the cardiac function since new lymphatic vessel formation reduces secondary edema and facilitates pumping during the systole and diastole. Decreased hypertrophy leads to diminished remodeling and cardiomyocyte death due to stress with consequent reduced interstitial scarring (Yoon et al., 2003; Henri et al., 2016; Vieira et al., 2018).

### Lymphangiogenesis and the Role Lymphatic Endothelial Cells

New lymphatic vessels in any injury will enhance the removal of cell debris and contribute to the resolution of the inflammation recruiting the immune cells back to the circulation (Yoon et al., 2003; Henri et al., 2016; Vieira et al., 2018). This aspect is extremely important to enhance tissue repair, but what are the consequences to the oxygen distribution and the consequential vascularization? It is yet to be defined whether lymphangiogenesis enhances later vascular formation and if lymphatic endothelial cells themselves influence the physiology of the neighboring cells when they, like all the other cells in the tissue, are targeted by inflammatory signals. The improvement from the lymphangiogenesis comes from the capacity to create a more lymphatic vessel, but the lymphatic cells, as an entity, will receive stimuli and communicate their activation to other cells as well. The major lymphatic endothelial cell markers are as follows: Lyve-1, the receptor of hyaluronic acid, Prox-1, the major transcription factor for the lymphatic fate, VEGFR-3, vascular endothelial growth factor for the VEGF-C, and podoplanin (Yang et al., 2012). Mature lymphatic endothelial cells have a heterogeneous origin (Klotz et al., 2015; Norman and Riley, 2016) and derive from already resident lymphatic endothelial cell (Ratajska et al., 2014; Klotz et al., 2015), venous endothelial cells (Ratajska et al., 2014; Norman and Riley, 2016), angioblasts (Nicenboim et al., 2015), or pluripotent stem cells (Salven et al., 2003; Lee et al., 2015). Furthermore, it has been described that venous endothelial cells and pericytes can differentiate into lymphatic endothelial cells by upregulating expression of the major lymphatic transcription factor Prox-1 (Petrova et al., 2002; Hirakawa et al., 2003; Yee et al., 2017); it has been published that altering the level of Prox-1 expression during the embryonic, post-natal, or adult stages can reprogram the lymphatic endothelial cells phenotype to become blood endothelial cells (Petrova et al., 2002; Groger et al., 2004; Yang and Oliver, 2014). On the other hand, blood endothelial cells can be transcriptionally reprogrammed by overexpression of PROX1 *in vitro*, resulting in upregulation of lymphatic markers (Petrova et al., 2002; Hirakawa et al., 2003; Yang and Oliver, 2014; Yee et al., 2017). In the cardiac scar tissue after MI, perivascular PDGFRβ-positive cells during the chronic phase of myocardial remodeling expressed both Prox-1 and podoplanin, which suggests that pericytes can differentiate into lymphatic endothelial cells and not only into fibroblast and contribute to the lymphatic vasculature (Cimini et al., 2017). Pericytes are another category of cells that have a lot in common with podoplanin-positive cells or are actually pericytes that acquire podoplanin during inflammatory conditions (Cimini et al., 2017).

## Pericytes: Sentinels of Few, Precursors of Many

Pericytes belong to the big family of adult mesenchymal progenitor cells and have potential to self-renew and differentiate into multiple mesenchymal cell types (Farini et al., 2014; Birbrair et al., 2015). Pericytes play a major role in the maintenance of blood vessel walls (Diaz-Flores et al., 2009), promote angiogenesis, vasculogenesis, tissue regeneration and repair, diapedesis of immune cells, and fibrogenic responses (Diaz-Flores et al., 2009; Wong et al., 2015). Pericytes have been studied mostly for their location and morphology; they can be spindle shaped, stellate, and with fingers-like projections surrounding vessels, and for these reasons reside in all the organs (Proebstl et al., 2012; Hall et al., 2014). They have been hypothesized to be precursors of mesenchymal stem cells (MSCs) since MSCs are anatomically found near the vasculature and perhaps can be isolated from most tissues around the body (Caplan, 2008; Feng et al., 2010; Crisan et al., 2012; Wong et al., 2015). Pericytes are recruited from endothelial cells through PDGF that binds PDGFRβ, highly expressed on pericytes, and on the other side angiopoietin-1 released from pericytes, mediates the binding between the two types of cells via a Tie2 receptor (Sundberg et al., 2002; Bjarnegard et al., 2004; Cai et al., 2008; Birbrair et al., 2014). PDGFRβ is probably the most well-known marker for pericytes found throughout, but there are others that are not uniquely found on pericytes and are often dynamically expressed (Armulik et al., 2005; Caplan, 2008; Crisan et al., 2008) or shared with endothelial cells, smooth muscle cells and MSCs (Crisan et al., 2012). Pericytes do not express hematopoietic and endothelial cells markers such as CD45, CD177, CD34, CD133, and CD31 (Crisan et al., 2008; Chen et al., 2015; Wong et al., 2015), but they are always positive for CD146, desmin, and 3G5 (Crisan et al., 2008). They irregularly express PDGFRα, NG2, and αSMA based on the vessel that they surround (Crisan et al., 2012; Chen et al., 2015). It has been described that, both *in vivo* and *in vitro*, pericytes express CD105, CD73, CD90, and Sca-1 as well as other well-known MSCs markers; on the other side also MSCs share with pericytes the expression of NG2, 3G5, CD146, PDGFRβ, and αSMA (Chen et al., 2015). Due to the anatomical position, morphology, and markers it is very difficult to determine whether these cells represent counterparts of the same population unless MSCs are isolated from bone marrow (Bautch, 2011; Crisan et al., 2012; Lin and Lue, 2013; Bobryshev et al., 2015; Klein, 2016).

### Pericytes and Podoplanin

Besides regenerative capacity, pericytes can contribute to perivascular and infiltrative fibrosis due to their plasticity (Thomas et al., 2017; Buhl et al., 2020); in fact, under hypoxia PDGFRβ-positive cells undergo endothelial to mesenchymal transition. Furthermore, it is well known that perivascular tumors derive from activated pericytes and the cancer tissue is highly positive to PDGFRβ (Palmieri et al., 2013). Recently it has been described that kidney fibrosis is characterized by the expansion of PDGFRβ-positive cells, and the inhibition of the PDGFRβ reverses the fibrosis (Wakisaka et al., 2019; Buhl et al., 2020). Only a few tissues where pericytes-derived fibrosis is disadvantageous to organ function have been investigated for podoplanin-positive cells in the fibrotic area (Song et al., 2014; Kolar et al., 2015; Lowe et al., 2015a; Cimini et al., 2019; Meng et al., 2020). This connection leads to the fact that pericyte and podoplanin expression may be very connected (Cimini et al., 2017). In the central nervous system, in fact, pericytes accumulate after tissue injury and release collagen (Birbrair et al., 2014), reducing pericyte-derived scar promotes recovery after spinal cord injury (Dias et al., 2018). On the other hand, transplantation of allogenic pericytes improves myocardial vascularization after MI (Alvino et al., 2018) due to the regulation of the endothelium in angiogenesis (Caporali et al., 2017).

## Mesenchymal Stem Cells and Their Contribution to Tissue Homeostasis

A very thin line separates pericytes from MSCs, probably they derive from the same group of cells with a distinctive evolution in the tissue. Perivascular niche-derived MSCs must be CD105-, CD73-, and CD90-positive (Bautch, 2011; Crisan et al., 2012; Lin and Lue, 2013; Shammaa et al., 2020) and negative for CD45, CD34, CD14, CD11b, CD79a, CD19, and HLA-DR (Crisan et al., 2008; Shammaa et al., 2020). It is very important that MSCs adhere to plastic when cultured *in vitro* and undergo the tri-lineage differentiation into osteoblasts, chondrocytes, and adipocytes (Wong et al., 2015). Murine MSCs have been largely described to express also Sca-1, CD146, PDGFRα, and PDGFRβ (Feng et al., 2010). In the bone marrow, a population of Sca-1-positive cells named sinusoidal endothelial cells express podoplanin and contribute to the maintenance of hematopoietic stem cell nice (Xu et al., 2018). Podoplanin-positive cells described in the heart share with MSCs the PDGFRα expression (Noseda et al., 2015; Cimini et al., 2017) since PDGFRα-positive MSCs as well as pericytes reside in the heart (Noseda et al., 2015; Beltrami and Madeddu, 2018), it could be that these two cell populations acquire podoplanin after injury in a time-dependent manner (Cimini et al., 2017). PDGFRα in the heart has been described to be associated with Sca-1 in cardiac progenitor/stem cells; these cells showed cardiomyocyte, endothelial and smooth muscle lineage potential after grafting and augmenting the cardiac function (Noseda et al., 2015). Specifically, PDGFRα demarcated the clonogenic/cardiogenic Sca-1 stem/progenitor cell (Noseda et al., 2015). As is the case with mesenchymal stem cells, mesenchymal stromal cells also express PDGFRα. The International Society for Cell and Gene Therapy (ISCT) has recently taken a position and made a statement to clarify the nomenclature because the two types of cells share most of the markers, especially when isolated from tissues different than bone marrow (Viswanathan et al., 2019). The ISCT continues to support the use of MSCs for both types of cells but supplemented with the tissue-source origin of the cells, intending to call them mesenchymal stromal cells, unless rigorous evidence for stemness can be supported by *in vivo* and *in vitro* data (Viswanathan et al., 2019). Competent MSCs have multiple therapeutic utilities due to their properties, are immune privileged due to the low expression of MHC I/II, and are thus used in immune-based pathologies, produce an anti-neoplastic agent, induce anti-tumor immunity, and stimulate, through differentiation or paracrine factors, tissue regeneration (Shammaa et al., 2020). In the heart, MSCs stimulate cell-specific regenerative mechanisms after MI depending on the site of the niches; niches were detected intramyocardially in cell clusters and characterized by positive expression of vimentin, CD29, CD44, CD105, and PDGFRα (Klopsch et al., 2017). PDGFRα-positive cells only have been found in the heart in the epicardium, myocardium, and endocardium; *in vitro* differentiation of cardiac PDGFRα-positive cells generates a significant number of smooth muscle cells and endothelial cells only (Chong et al., 2013). These data suggest that cardiac MSCs PDGFRα-positive cells predominantly contribute to the vascular and mesenchymal compartments.

### Mesenchymal Stromal Cells: A Heterogeneous Cell Population

Like MSCs, mesenchymal stromal cells have also been identified in the heart and specifically in both ventricles (Stadiotti et al., 2020). Histological analysis showed a greater percentage of stromal cells in the right ventricle versus the left one; cardiac mesenchymal stromal cells from the right ventricle show the same surface markers as the cells isolated from the left ventricle (Stadiotti et al., 2020). There is a very thin line that separates MSCs from mesenchymal stromal cells, and in the heart, a dynamic flux of cardiac stromal cells has been described, which is much that like when MSCs express PDGFRα (Farbehi et al., 2019). During MI, the population of PDGFRα-positive mesenchymal stromal cells is highly enriched in the ischemic area at 3 and 7 days post-surgery (Farbehi et al., 2019). The PDGFRα-positive stromal cells isolated after MI showed at the single-cell RNA sequencing either pro and anti-fibrotic characteristics, thus mesenchymal stromal cells follow a non-linear differentiation in myeloid or fibroblast lineage after injury (Farbehi et al., 2019). Based on these data, the mesenchymal stromal/stem cells belong to a heterogeneous population, which contributes to both regeneration and tissue homeostasis (repair) based on the stemness of the niches, and they share the expression of PDGFRα and can be pharmacologically targeted to manage fibrosis (Usunier et al., 2014; Klimczak and Kozlowska, 2016).

### Podoplanin and Mesenchymal Cells

There is no evidence to support which type of PDGFRα-positive cell acquires podoplanin, be it MSCs or the mesenchymal stromal cells, at least in the heart. But it is very important to understand whether the activation of MSCs or mesenchymal stromal cells in fibrosis is marked by podoplanin. Recently, Forte et al. identified differences in epicardial- and endocardial-derived fibroblast of two different types of inbred mice based on the frequency of the left ventricle rupture after MI, and they found that the interstitial cell activation is crucial for the scar formation and organ survival after injury (Pinto et al., 2016; Forte et al., 2020). Based on these data, and the behavior of PDGFRα-positive cells, it is possible to corroborate the idea that either MSCs or mesenchymal stromal cells acquire podoplanin after injury (Cimini et al., 2017). Ward et al. (2019), recently described the upregulation of podoplanin in mesenchymal stromal cells and showed the functional consequences of podoplanin expression on the migration of mesenchymal stromal cells and their interaction with platelets. In a co-culture system using porous *trans-*wells, podoplanin-expressing mesenchymal stromal cells were able to create microthrombi after capturing platelets, and treatment with recombinant soluble CLEC-2 inhibited the aggregation (Ward et al., 2019). Similar phenomena have been described in atherosclerotic lesions (Hatakeyama et al., 2012) and calcification of aortic valves (Napankangas et al., 2019), where mesenchymal cells have been described to enhance the podoplanin expression; specifically, MSCs have been the most mesenchymal cells isolated from calcified and inflamed aortas. Their origin has often been confirmed *in vitro* to show three lineage potentials (Ciavarella et al., 2017). Notably, MSCs like pericytes and podoplanin-positive CAFs showed an extracellular-vesicle mode of communication (Valente et al., 2015) that could participate in the mechanism of arterial calcification (Zazzeroni et al., 2018). MSCs can be histologically confused with another type of interstitial cells called telocytes, which share with MSCs the expression of PDGFRα but differ from MSCs by high expression of CD34 (Kucybala et al., 2017).

## Telocytes and Podoplanin

Telocytes are enigmatic interstitial cells with a very distinctive morphology and a small body with long extensions named telopodes; they have been characterized by specific markers, tissue localization/geometry, and physiological function (Kucybala et al., 2017). Projections are features that telocytes have in common with CAFs where they are called invadopodia (Martin-Villar et al., 2015), and MSCs and pericytes, named prolongations (Thomas et al., 2017), and lymphatic endothelial cells (Rusu and Hostiuc, 2019). Telopodes from telocytes, except for neural axons, are the longest structures in the body: they branch and create a pattern (Popescu and Faussone-Pellegrini, 2010; Varga et al., 2016). Telocytes are located in almost all the organs, heart included, in the interstitium, extra epithelial space, between functional elements like arteries and nerves (Cretoiu et al., 2014; Rusu et al., 2014; Mirancea, 2016), and can be ultrastructurally confused with lymphatic endothelial cells due to the lack of basal lamina (Rusu and Hostiuc, 2019). Telocytes are specifically positive for CD34, PDGFRα, PDGFRβ, c-Kit, Sca-1, CD29, vimentin, and α-SMA (weak) (Chang Y. et al., 2015; Diaz-Flores et al., 2016; Faussone-Pellegrini and Gherghiceanu, 2016) and negative for CD45 (Bei et al., 2015a). In contrast, fibrocytes, bone-marrow-derived MSCs, doubly express CD34 and CD45 (Keeley et al., 2009; Piera-Velazquez et al., 2016). It is very difficult to differentiate telocytes from other types of cells, and the most appropriate marker to use to recognize them is the CD34 since MSCs, pericytes, fibroblasts, and neurons are negative for it (Bei et al., 2015b; Mirancea, 2016). These cells constitute a part of podoplanin-positive cells after 15 days of MI (Cimini et al., 2017). Telocytes are well known to communicate with other cell types, specifically in the heart with cardiac stem cells, with stromal synapses, point contacts, nanocontacts (Manole et al., 2011; Faussone-Pellegrini and Gherghiceanu, 2016; Popescu et al., 2016) like MSCs, and pericytes via extra cellular vesicles and exosomes (Fertig et al., 2014; Faussone-Pellegrini and Gherghiceanu, 2016).

### Telocytes and Cardiac Regeneration

The contacts built by telocytes have a mechanical function and allow intercellular communication as an exchange of information (Faussone-Pellegrini and Gherghiceanu, 2016) beyond the well-known paracrine communication (Manole et al., 2011; Edelstein et al., 2016). Physiologically, telocytes support cardiac growth, regeneration, renovation of connective tissue, and repair due to the unique communication with cardiac stem and progenitor cells (Popescu et al., 2015; Diaz-Flores et al., 2016; Li Y. et al., 2016). During the pathological condition, in the heart, the number of telocytes decreases; consequently cardiac stem cell niches are impaired with a consequent increase in fibrosis as a replacement for the loss of telocytes, cardiac stem cells, and signalization between telocytes and fibroblasts (Richter and Kostin, 2015; Kostin, 2016). The telocyte-associated diseases are named telocytopathies (Ibba-Manneschi et al., 2016; Varga et al., 2019). Telocytes are also found along the vascular system, are located in proximity of MSC perivascular niches, and are positive for CD34 and PDGFRβ; they connect cells within each other and support angiogenesis (Suciu et al., 2010; Cantarero et al., 2011; Zhang et al., 2015; Boos et al., 2016). Isolated rat telocytes for CD34/PDGFRα continue to express these two markers also *in vitro* together with vimentin (Li Y. Y. et al., 2016) as well as isolated murine telocytes (Chi et al., 2015) without losing their typical morphology. *In vitro* murine telocytes secretory profile has been investigated with and without the presence of cardiac stem cells; isolated telocytes express IL-2, IL-6, IL-10, IL-13, VEGF, MIP-1α, and MIP-2 when cultured alone, and, in the presence of cardiac stem cells, MIP-1α and MIP-2 increased (Albulescu et al., 2015). Like cytokines, they release extracellular vesicles were loaded with microRNAs to cardiac stem cells (Cismasiu and Popescu, 2015). In the heart, CD34-positive/CD45-negative cells co-stain with podoplanin during the chronic phase of MI (Cimini et al., 2017).

## Conclusion

Podoplanin is not expressed physiologically in the heart except in lymphatic endothelial cells and pericardial area, which means that probably it can be acquired over time by pericytes, MSCs, and telocytes that reside in the heart and that are already positive for PDGFRβ, PDGFRα, and CD34. Assimilating this information and interpreting the appearance of podoplanin over time after MI, we can speculate that at 2 days after MI, PDGFRα-positive cells acquire podoplanin, and then at 2 weeks, the PDGFRβ- and then the CD34-positive cells become podoplanin positive. It could be that first MSCs, then pericytes and in the end telocytes, express this glycoprotein under inflammatory conditions from the acute to the chronic phase of MI and thus orchestrate the cell-to-cell communication with monocyte, endothelial cell, and progenitor cell niches. In order to solve this mystery, lineage tracing is needed to understand if these determined categories of cells are the ones that acquire this glycoprotein, and, if they do, what the major signal that contributes to the podoplanin expression is. Furthermore, how this expression changes the biology of the cells after podoplanin acquisition and whether this acquisition results in loss of some specific markers are things that remain to be assessed. For example, podoplanin-positive cells do not express c-Kit, vimentin and α-SMA, CD34-positive telocytes probably lose the c-Kit after the expression of podoplanin (Zhou et al., 2015; Cimini et al., 2017; Hostiuc et al., 2018), or PDGFRβ- and PDGFRα-positive cells activate their PDGFRs. The thin line between expression and activation is likely shown by the acquisition of podoplanin; functional studies of podoplanin-positive cells will help to understand the mechanism by which podoplanin-positive cells contribute to tissue homeostasis in health and disease (Frangogiannis, 2019). Podoplanin-positive cells do not have to be villains because each subpopulation actively contributes also to the regeneration of the necrotic area; telocytes are extremely important for the maintenance of cardiac stem cells niches, if they acquire podoplanin, it will be interesting to understand if one of the consequences is a detrimental communication between telocytes and cardiac stem cells (Bei et al., 2015a, 2016). In conclusion, a linage tracing (Pinto et al., 2016) and a single cell transcriptome profiling (Skelly et al., 2018) of all the subgroups of podoplanin-positive cells is needed to better understand where podoplanin cells come from. Understanding the origin of podoplanin-positive cells and their evolution can help to modulate the cell-to-cell and paracrine communication during inflammation, based on their role in physiological conditions, and may open a new line of investigation extending beyond known pro-reparative properties.

## Author Contributions

All authors listed have made a substantial, direct and intellectual contribution to the work, and approved it for publication.

## Conflict of Interest

The authors declare that the research was conducted in the absence of any commercial or financial relationships that could be construed as a potential conflict of interest.
